# Benefits and damages of the use of touchscreen devices for the development and behavior of children under 5 years old—a systematic review

**DOI:** 10.1186/s41155-020-00163-8

**Published:** 2020-10-31

**Authors:** Bruno Rocha, Cristina Nunes

**Affiliations:** 1 University of Algarve, Faro, Portugal; 2 Psychology Research Centre (CIP), University Autónoma de Lisboa, Lisboa, Portugal

**Keywords:** Screen time, Infant, Child, preschool

## Abstract

**Aim:**

Several health organizations have been expressing concern about the amount of time children spend using electronic devices and about the benefits and damages of the use of touchscreen devices, such as smartphones or tablets, for the development and behavior of children under the age of 5. A systematic review was carried out in order to understand the impact of touchscreen device usage toward children’s development and behavior under the age of 5.

**Methods:**

Using the PRISMA method, from a total of 6314 studies found in online databases, searched in English, between 01/01/2000 and 01/10/2018, 11 studies were selected for analysis.

**Results:**

The results revealed that, in children under the age of 5, the damages of the use of touchscreen devices are superior to the benefits that may result, especially when there are more hours of screen time. More importance is given to the quality of the child-adult relationship and not to the use of touchscreen devices. Nevertheless, some studies emphasize some aspects that may reduce the negative effects, such as moderate use, less screen time, parental monitoring, and viewing educational programs in an academic environment.

**Conclusions:**

Guidelines that should be given to parents about the use of touchscreen devices by children are discussed. The limitation of this study was the difficulty in finding studies directed to the desired age and type of electronic device. This may be taken as a potential cause of bias.

## Introduction

The way electronic devices and the internet have been integrating our lifestyle is a current concern. It is inevitable that this evolution affects the daily experiences of children. In addition to the interactive television and thematic children’s channels (Ponte, Simões, Baptista, Jorge, & Castro, 2017), the use of touchscreen devices with internet connection such as smartphone devices or tablets is increasing (Brito & Ramos, 2017; SBP—Brazilian Society of Pediatrics, 2016).

It is recommended that the use of electronic devices should be limited to children and be proportional to the ages and stages of cerebral, mental, cognitive, and psychosocial development (SBP, 2016). Both the American Academy of Pediatrics (AAP) and the Brazilian Society of Pediatrics (SBP) advise against using electronic devices until the age of 2 (AAP Council on Communications and Media, 2016; SBP, 2016), noting that despite the benefits, the damages that affect children are bigger. More recently, the AAP has advised that from 18 months to 2 years old, parents who wish to begin the introduction of technology should always do it with supervision, properly choosing the applications, and avoiding the self-use by children. It also states that before 18 months, the only type of screen exposure tolerated is video chatting.

After 2 years old, exposure to the media is not recommended for more than 1 h per day (AAP Council on Communications and Media, 2016). The World Health Organization issued strategic recommendations to reduce children's sedentary lifestyle, which were to not expose technology to children under the age of 2 and to allow only 1 h a day of screen time for children who are between 2 and 5 years old (WHO, 2019).

Despite the above recommendations, the use of technology among children is common. Taylor, Monaghana, and Westermanna (2018) pointed out that screen media exposure in children under the age of 3 is high, instead of completing educational activities. Approximately half of the 2-year-old population access the Internet occasionally, increasing this number, frequency, and autonomy with their age (Howe et al., 2017). Also, children under the age of 2 frequently use electronic devices (Palha et al., 2019; Taylor et al., 2018). Despite the fact that television is the most widely used electronic device (Adisak, Chiranuwat, Pongtong, & Sakda Arj-Ong, 2018; Ponte et al., 2017), touchscreen devices are increasingly used by children (Ponte et al., 2017). In children between the ages of 2–4 years old, it was observed that the most efficent way of using touch screen devices is through children and parents using them together (Guedes et al., 2020).

Although there are studies that show some benefits and few damages of the use of electronic devices, the growing concern of screen usage is accompanied by the evidence that they have negative effects on the health and development of children, being associated with child obesity, sleep disorders, concentration disorders, as well as taking time from other important activities such as reading or creative play (Carter, Rees, Hale, Bhattacharjee, & Paradkar, 2016; Howe et al., 2017; Huber, Yeates, Meyer, Fleckhammer, & Kaufman, 2018; Shukla & Jabarkheel, 2019). Brockmann et al. (2016) states that the use of electronic devices before bed is related to more night terrors and sleep problems. Also screen time for more than 2 h a day was shown to be closely related to the sedentary behavior of preschool children (Guerra et al., 2019). There is also concern about the negative relationship of socialization and school achievement, toward older ages and security issues (SBP, 2016). Another disadvantage reported in the literature is the delay in language and cognitive development (Adisak et al., 2018). Madigan, Browne, Racine, Mori, and Tough (2019) observed that higher levels of screen time at 24 and 36 months are related with poor performance on developmental screening tests.

The greater use of screens tends to be related to low academic and social conditions, unemployment, sedentarism of the family, single parenting, maternal depression, the existence of television in the children’s room, the lack of rules on the usage of the television, and the greater use of television by parents (Howe et al., 2017). Also, older mothers are related with exposing their children later to technology/electronic devices (Palha et al., 2019). Electronic devices appear as a *coping* strategy for parents’ frustration with children’s behavior, with a calming effect, and as a facilitator of daily routines and childcare. These strategies are commonly used in children who cry the most or when parents feel that children have bad temperament (Ponte et al., 2017; Radesky, Peacock-Chambers, Zuckerman, & Silverstein, 2016). The American Academy of Pediatrics recognizes that there are certain times when the use of the media can be useful as a strategy to reassure the child, such as performing medical procedures or during air travel. However, it is revealed that the use of this strategy may lead to problems such as the limitation or the inability of children to develop their own emotional regulation mechanisms (AAP Council on Communications and Media, 2016).

Many parents are not aware to the negative effects of screen exposure toward their children (Adisak et al., 2018), even considering that the use of educational applications in the smartphone may be beneficial (Guedes et al., 2020; McCloskey et al., 2018). It cannot be ignored that there is a widespread use of electronic devices and the promotion of healthy behaviors requires the integrated management of technologies in the daily routine (Patrão & Sampaio, 2016; SBP, 2016). Magalhães, Fernandes, Mendes, and Martins’s (2015) results suggest that the use of digital technology can increase family interventions’ cost/benefit ratios, creating a broader public and promotion health impact.

Brito & Dias (2019) reinforces the importance of the type of use and parental mediation. Parents with a more positive perspective about the pedagogic use of technology tend to encourage children using and perceiving electronic devices as an important tool for learning. On the other hand, parents who hold a less positive view about digital media do not encourage their use, not even for pedagogic purposes, frequently restricting screen-time, and therefore their children use the devices for limited time and mostly for leisure activities.

Despite the major frequency of children watching television in the children’s screen time, this study focuses on the use of touchscreen devices, such as the smartphone and the tablet being the ones more relevant in the literature. This systematic review aims to know the benefits and damages of the use of touchscreen devices toward the development and behavior of children under 5 years of age. The research will aim to answer the following specific questions: Are touchscreen devices beneficial or harmful to the development of children under the age of 5? Are touchscreen devices beneficial or harmful to the behavior of children under the age of 5? Are there any factors that influence the beneficial or harmful effects of using touchscreens on children, or is the effect of the use the same despite the context?

## Methodology

### Formulation of the question

This systematic review was made according to the PRISMA recommendations.

The formulation of the question was defined through the PICO strategy (Population, Intervention, Comparison, Outcomes (results)). Thus, the study population corresponds to children under the age of 5 (P); the intervention studied is the usage of touchscreen devices (I). In this study, there was no comparison between standard intervention and other interventions (C) and the expected results are the effects of the use of touchscreen devices in health and development of children (O). Thus, the guiding question of the study was: For children under the age of 5 (P), does the use of touchscreen devices (I) have positive or negative effects on their development and behavior (O)?

### Eligibility criteria

The following inclusion criteria was considered: (a) studies published in English; (b) studies in children under 5 years of age or until the pre-school age; (c) use of touchscreen devices such as smartphones or tablet computers; (d) objectively demonstrate an advantage or disadvantage of the usage of these devices; (e) studies published between the dates of 01-01-2000 and 01/10/2018; and (f) peer-reviewed studies in scientific journals.

For the exclusion criteria, the following were not considered: (a) articles related to children with specific pathologies; (b) articles in which part of the participants were older than those included in the inclusion criteria and the results were not discriminated by age; and (c) studies without reference to ethical procedures.

Exclusion criteria (b) was considered relevant because there were several articles that studied the use of electronic devices and specific applications in children with diseases like diabetes, cancer, or autism, among others. The targeted use of technology in specific pathologies, and the achievements in children was not the aim of this study, so the objective of these exclusion criteria was to remove studies with a therapeutic purpose in specific groups and contexts, reducing bias.

### Research strategy

The studies for this research were obtained through the search engines EBSCOhost—Research Databases and B ON in the following electronic databases: CINAHL Complete; MEDLINE Complete; Nursing & Allied Health Collection: Comprehensive; Cochrane Central Register of Controlled Trials; Cochrane Database of Systematic Reviews; Cochrane Methodology Register; Library, Information Science & Technology Abstracts; MedicLatina; ScienceDirect; ERIC; Directory of Open Access Journals; Supplemental Index; IEEE Xplore; Digital Library; SciELO; Science In Context; Digital Access to Scholarship at Harvard (DASH); and Literature Resource Center.

The identification of the terms to elaborate the research in the electronic databases resulted from a preliminary research of studies related to the same subject, grouping synonyms for the following terms: child; electronic devices; benefits and losses. Some terms were also selected relating to frequent diseases excluded in the study, as well as other frequent words not considered in order to reduce the research to the inclusion criteria.

**Fig. 1:**
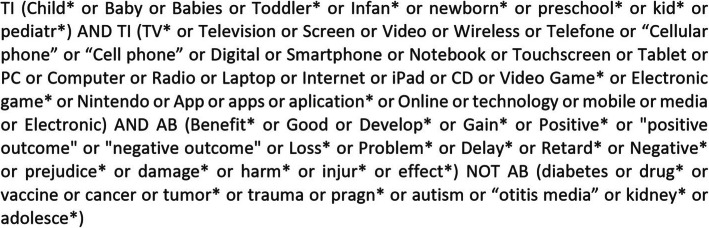
Truncated search strategy

Thus the research was accomplished with the truncated search strategy presented below (Fig. 1).

### Data collection

**Fig. 2:**
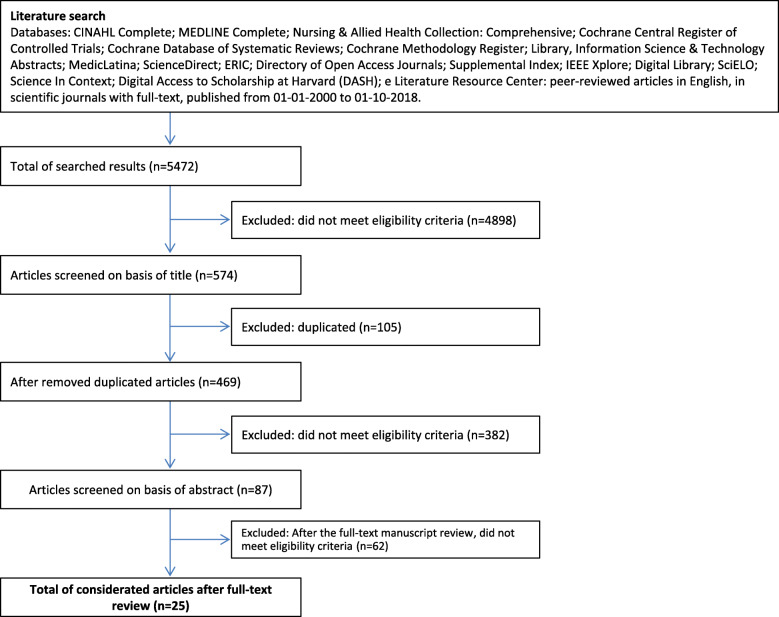
Systematic review procedure

The search and identification of articles, selection, and collection of data were paired up by the two authors. The studies were sorted by recent, peer-reviewed, scientific journals, and with full-text available, obtaining a total of 6314 studies. After this first selection, the articles related to the theme were selected by their title. From this, the duplicate articles were removed, followed by the abstract analysis. After reading the abstract from 469 studies, 25 were selected for full-text reading, with completing the selection process with a total of 11 studies for analysis (Fig. 2).

## Results

### Extracted variables

From the analysis of the selected studies, the following variables were extracted: the date of publication, the number of sample participants, the age of the participants, the type of study, the main data analysis plan, the electronic devices under the study, the effect of the electronic devices in children, the country where the study was conducted, and the main findings.

### Participants

Of the 11 articles reviewed, 7 were mostly related to the effects of electronic devices on cognitive development, 3 with child behavior and temperament, and 1 with various subjects in general.

### Results presentation

Table 1 shows the variables and the results of each study analyzed.

**Table 1:** Characteristics of the questionnaires and the samples

Authors and Year of Publication			Tipe of study			Analysis plan	Devices								
	Sample (n)	Age	Transversal	Longitudinal	Experimental		Tablet	Smartphone	Games	Computer	Internet	TV	Effect in studie	Country	Main conclusions
Mendelsohn, AL, Brockmeyer, CA, Dreyer BP, Fierman, AH, Berkule-Silberman, SB, & Tomopoulos, S. (2010).	253	6 months and 14 months		●		Hierarchical Multiple Linear Regression	●	●		●		●	Cognition	USA	Talking and interacting with children during the use of electronic devices with educational programs (not others) improves children's acquisition of language.
Papadakis, S., Kalogiannakis, M., & Zaranis, N. (2018)	265	x = 62 month (SD = 5.5)			●	ANOVA	●			●			Cognition	Greece	The use of computer or tablet with appropriate programs and software in a school context promote more learning.
Vatalaro, A., Culp, AM, Hahs-Vaughn, DL, & Barnes, AC (2018)	63	3 to 5 years			●	ANOVA	●	●					Cognition	USA	The use of well selected applications to improve vocabulary in a school context is better than the free use of electronic devices.
Huber, B., Yeates, M., Meyer, D., Fleckhammer, L., & Kaufman, J. (2018)	96	x = 36.3 month (SD = 7)	●			ANOVA	●	●				●	Cognition	Australia	The type of viewing interferes with the cerebral executive function of the children. Using an educational application makes the child more capable of delaying less quick gratification for a longer but better one, than seeing a normal cartoon. Between an educational application, an educational TV show or a normal cartoon, the application is the kind of media that has the best effect on memory. This study shows that the interaction and type of content is more important than merely screen time.
Taylora, G., Monaghana, P., & Westermanna, G. (2018)	131	x = 44.37 month (SD = 2.32) x = 20.44 month (SD = 2.48)	●			ANOVA	●	●				●	Cognition	UK	There is no evidence that suports that screen time is positive or negatively related with children's vocabulary. There is a positive relationship between vocabulary and the time spent reading with children.
Bedford, R., Saez of Urabain, IR, Cheung. CHM, Karmiloff-Smith, A., & Smith, TJ (2016)	715	6 to 36 months	●			ANOVA	●						Cognition	UK	No negative relationship was found between the use of the touchscreen and the reaching of gross motor development. A positive relationship was found between use of the touchscreen and the attainment of fine motor skills.
Tomopoulos, S., Valdez, PT, Dreyer, BP, Fierman, AH, Berkule, SB, Kuhn, M., & Mendelsohn, AL (2007)	70	3 to 5 years	●			Multiple Regression	●		●	●		●	Cognition	USA	Increased exposure to non-educational content for children leads to less time for teaching and reading activities at home, which can negatively influence development.

## Discussion

The literature described the use of electronic devices as harmful in various aspects of children’s health; some positive effects can also be found (AAP Council on Communications and Media, 2016; SBP, 2016). These observations met the results verified in this systematic review.

Papadakis et al. (2018) stated that the use of computers, especially tablets, by children, using an appropriate educative software combined into the children’s daily routine, promotes their learning (pretest: *F* (2, 362) = 9.75, *p* < .001 and post-test: *F* (2,361) = 26.13, *p* < .001). The scores of the tablet group increased from *M* = 19.34 (*SD* = 6.02), to *M* = 25.26 (*SD* = 6.52) in the post-test. The AAP Council on Communications and Media (2016) adds to the importance of adapting content to children’s age. Other authors reinforced that learning in a real context is more beneficial than in any electronic device, even when applications are designed for that purpose (Huber et al., 2018). Magalhães et al. (2015) suggested that digital technology can also increase family interventions’ cost/benefit ratios.

However, the type of content in digital media used is a powerful determinant, since it is less detrimental to spend more time with an educational content than less time with non-educational content (Huber et al., 2018). The benefits of acquiring vocabulary exist when the contents of the programs are educational and when they are accompanied by the parents (Mendelsohn et al., 2010). This practice is common by parents who believe in the benefits of technology use (Guedes et al., 2020). Vatalaro et al. (2018) demonstrated the benefits of using a scaffolding-like vocabulary application rather than any open-ended vocabulary application. Taylor et al. (2018) suggest that teaching and reading activities act as a beneficial agent for learning. These authors also devalue the screen time where they did not find benefits or damages in their use. However, Tomopoulos et al. (2007) concluded that increased exposure to non-educational content for children leads to less time for teaching (*SR* = − 0.27, *P* = 0.01) and reading (*SR* = − 0.24, *P* = 0.02) activities at home, which can negatively influence development.

Regarding the effect of electronic devices on temperament and behavior, no studies were found to demonstrate positive effects. Wu et al. (2014) revealed that problems related to children’s temperament and behavior tend to be related to longer screen exposure. These problems are aggravated with not educational and antisocial content of the screen exposure (*B*: 3.84, 95% CI: [1.66, 6.02], *p* < 0.01).

Zhao et al. (2018) suggests that the increase in screen time is related to lower psycho-social welfare and social behavioral problems. For example, the score of social behavior problem changes from 1.1, 95% CI: [1.0, 1.2] to 1.4, 95% CI: [1.3, 1.6] when the screen time increases from 1 to 2 h to more than 4. Poulain et al. (2018) relates the use of mobile phones with conduct problems (*b* = 0.55, *p* < 0.05) and attention disorders in the future (*b* = 1.10, *p* < 0.01). The reverse association was also verified by Poulain et al. (2018), as baseline higher scores in peer relationship problems are related to a greater use of the mobile phones in the future (OR = 1.58, *p* < 0.001).

These results are in agreement with Carter et al. (2016), Guerra et al. (2019), Huber et al. (2018), Howe et al. (2017), and SBP (2016), when they relate the use of electronic devices to sleep disturbances, concentration, socialization, and school achievement. Our results reinforce that behavioral and temperament problems tend to get worse when the time of exposure to technology increases. Akçay and Emiroğlu (2019) stated that motivational interventions can develop behavioral change in aggressive children, increasing auto-control and setting limits on media use.

Nevertheless, studies with no significant relationship were found in this aspect, as shown by Tansriratanawong, Louthrenoo, Chonchaiya, and Charnsil (2017), which simply did not find any significant relationship between screen time and behavioral problems in children. Sugawara et al. (2015), despite the damages shown above, confirms that technology/screen time is not related to problems of conduct or attention in children, as suggested by Poulain et al. (2018).

Poulain et al. (2018) calls into question the orientation of the predictive effect of temperament problems, since children with basic interpersonal relationship problems tend to increase the likelihood of using all of these devices over time. Thus, Zhao et al. (2018) states that screen time is mediated by variables such as interaction with parents, body mass index, and sleep quality. Sugawara et al. (2015) states that it is the characteristics of the families that lead to increase or not the exposure to the screens. Howe et al. (2017) also pointed out that electronic devices are used as a calming device for children’s behavior and are widely used by parents as an entertainment strategy to perform household chores at home. Ponte et al. (2017) and Radesky et al. (2016) reinforce that this strategy tends to be used more by depressed mothers and mothers of children that cry more or that their behavior is considered to be more agitated by them.

The American Academy of Pediatrics also recognizes, though with caveats, that there are certain times when the use of the media can be useful as a strategy to reassure the child, such as performing medical procedures or air travel (AAP Council on Communications and Media, 2016).

As for the effect of electronic devices on physical activity, in younger children, the use of electronic devices does not improve children’s gross motor skills; however, the early use of touchscreen displays is related to faster acquisitions in fine motor skills (*r* = 0.16, *p* = 0.03) (Bedford, Saez de Urabain, Cheung, Karmiloff-Smith,, & Smith, 2016). Literature states that the use of electronic touchscreen devices in young children is related with increased body mass index in the future (Carter et al., 2016; Howe et al., 2017; Shukla & Jabarkheel, 2019).

The present articles have reported some limitations which may be considered a potential cause of bias.

First, some authors reported that the data collection methodology would require significant engagement with the parents, since it is necessary to recall diaries or opinions about their children’s behavior. These results may underestimate information or provide a socially desirable response (Mendelsohn et al., 2010; Poulain et al., 2018; Wu et al., 2014).

The second described limitation is the representativity of the sample. Studies with convenience samples or made with specific sociodemographic population may not be representative of the general population in terms of socio-economic status (Mendelsohn et al., 2010; Papadakis et al., 2018; Poulain et al., 2018; Tansriratanawong et al., 2017; Tomopoulos et al., 2007; Wu et al., 2014).

The third limitation of some studies is the selected methodology and the causality inference, by the use of observational and cross-sectorial studies instead of experimental and longitudinal methods (Mendelsohn et al., 2010; Papadakis et al., 2018; Tansriratanawong et al., 2017; Taylor et al., 2018; Tomopoulos et al., 2007; Vatalaro et al., 2018; Zhao et al., 2018).

The fourth limitation reported in some studies was the potential bias caused by the study design, like the duration of the intervention or the intervention of the data collection (Papadakis et al., 2018), or when family had more than one preschool child which may confound answers (Wu et al., 2014). Bedford et al. (2016) and Huber et al. (2018) recognize the limitation of the unknown status of each child using a touchscreen prior to the moment in analysis and did not report other aspects of development related with touchscreen use such as health problems potentially related with it.

Lastly, in some studies, the content of programs or media applications have not been analyzed, since it is an important variable in children learning or behavior (Taylor et al., 2018; Zhao et al., 2018).

About the limitations of our study center on the difficulty in finding studies directed to a specific age and type of electronic device, such as smartphones or portable electronic devices. The vast majority of studies that relate electronic devices to children’s health target older children. Even when the studies are in children who are less than 5 years old, the majority of the participants also tend to belong to the higher ages. The range of age of one of the selected studies goes up to 6 years old; however, it is a smaller part of the sample; this study was considerate for its relevance. Also, many studies approached the findings indiscriminately in children of various ages, despite significant developmental differences between them. Another limitation of this study is that electronic devices tend to be addressed in a clustered way, and does not isolate the touchscreen devices from conclusions, being together with other devices such as television or videogames. Thus, the effects of other electronic devices also end up being part of some results, which may be taken as potential cause of bias.

Although this study has the limitations mentioned above, we consider that it contributes to aggregate and make a state of the art about what is known about the advantages and disadvantages of the use of touchscreen devices by children under 5 years old.

## Conclusion

It can be concluded that the damages resulting from the use of touchscreen devices for the development and behavior of children under 5 years of age outweigh the benefits that may result.

More importance is given to the quality of the child-adult relationship and not to the use of touchscreen devices. However, this is a strong distraction factor that may limit the child’s participation in other types of activities. The most recent guidelines of the WHO are to promote the activity of children and avoid sedentary lifestyle, being one of the recommendations restrict the use of screens.

Non-educational, non-mediated, and antisocial content is harmful to children, as well as excessive time using screens. On the other hand, the benefits of electronic devices are observed more of the time only in school settings, with adult participation and specific software apps.

It is also argued that children with a more agitated, aggressive, crying temperament, spend more time with electronic devices as a distraction strategy in order to help parents deal with this situation. In view of the results, it will be desirable that in these cases, the use of electronic devices be adapted to the age, educational, and with the participation of the parents. There should always be parental involvement when children use electronic devices and the less time of use the best for them. In addition, educational content adapted to the child’s age should be promoted instead of non-educational or antisocial content.

A healthy and active family dynamic as well as literacy was shown to be a protective factor of the screen time and the damages that come from a sedentary life. In a society where it is inevitable that children coexist around various types of electronic devices, the development of varied and outdoor activities is beneficial for child development, away from the easy tendency of excessive use of electronic devices.

For future recommendations, technology must be safely integrated into the growth of children, according to their age, and it is important that future studies demonstrate which contexts such electronic use may even be beneficial. Further studies are required to analyze the contents of the different touchscreen applications, in different ages and contexts.

## Data Availability

The studies that support the findings of this study are available in search engines EBSCOhost - Research Databases and B ON, and identified with the symbol asterisk “*” in the references.
